# Manipulation and prediction of spike morphology traits for the improvement of grain yield in wheat

**DOI:** 10.1038/s41598-018-31977-3

**Published:** 2018-09-26

**Authors:** Zifeng Guo, Yusheng Zhao, Marion S. Röder, Jochen C. Reif, Martin W. Ganal, Dijun Chen, Thorsten Schnurbusch

**Affiliations:** 1 Independent HEISENBERG Research Group Plant Architecture, Leibniz Institute of Plant Genetics and Crop Plant Research, 06466 Gatersleben, Germany; 2 Research Group Quantitative Genetics, Department of Breeding Research, Leibniz Institute of Plant Genetics and Crop Plant Research, 06466 Gatersleben, Germany; 3 Research Group Gene and Genome Mapping, Department of Breeding Research, Leibniz Institute of Plant Genetics and Crop Plant Research, 06466 Gatersleben, Germany; 4 TraitGenetics GmbH, 06466 Gatersleben, Germany; 5 Research Group Image Analysis, Leibniz Institute of Plant Genetics and Crop Plant Research, 06466 Gatersleben, Germany

## Abstract

In wheat (Triticum spp.), modifying inflorescence (spike) morphology can increase grain number and size and thus improve yield. Here, we demonstrated the potential for manipulating and predicting spike morphology, based on 44 traits. In 12 wheat cultivars, we observed that detillering (removal of branches), which alters photosynthate distribution, changed spike morphology. Our genome-wide association study detected close associations between carbon partitioning (e.g. tiller number, main shoot dry weight) and spike morphology (e.g. spike length, spikelet density) traits in 210 cultivars. Most carbon-partitioning traits (e.g. tiller dry weight, harvest index) demonstrated high prediction abilities (>0.5). For spike morphology, some traits (e.g. total and fertile spikelet number, spike length) displayed high prediction abilities (0.3–0.5), but others (e.g. spikelet fertility, spikelet density) exhibited low prediction abilities (<0.2). Grain size traits were closely correlated in field and greenhouse experiments. Stepwise regression analysis suggests that significantly associated traits in the greenhouse explain 35.35% of the variation in grain yield and 67.63% of the variation in thousand-kernel weight in the field. Therefore, the traits identified in this study affect spike morphology; these traits can be used to predict and improve plant architecture and thus increase yield.

## Introduction

The morphology of the inflorescence (termed the spike) of small-grain cereals is crucial in determining grain yield. The components of the spike (e.g. spikelets, which are the basic units of the inflorescence and contain florets, glumes, and lemma), also influence each other. The number and arrangement of these spike components affect spike length, spike weight, spike chaff (i.e. the non-grain biomass in the spike), grain number per spike, grain weight per spike, and spikelet number per spike, which all contribute to final grain yield per spike^1–3^. To produce varieties with the most efficient grain production in different environments, we need to know how to predict and manipulate spike morphology in wheat.

The formation of branches, termed tillers, affects grain number and grain weight, and is therefore closely related to yield^4–7^. Donald (1968) proposed that uniculm wheat, which does not form tillers, has the potential for greater yield compared to wheat with tillers^8^. Detillering can be used to study the relationship between tillering and the spike morphology factors discussed above. It has been reported that the increase in grain yield after tiller removal was mainly due to the increase of grain number per spikelet in wheat^9^.

In addition to tiller removal, other studies have removed spike components such as spikelets, florets, glumes and lemmas to assess their interactions with each other. For example, Pinthus and Millet (1978) found a small increase in grain number and a marked increase in grain weight in the remaining spikelets after spikelet removal in wheat^10^. They also showed that grain weight was determined by factors that affect grain volume^11^. Similarly, Wang *et al*. (1998) found that spikelet removal considerably increased grain number and grain weight per spikelet^12^. Removal of glumes and lemmas from wheat spikelets during the grain filling period resulted in less growth than in intact florets^13^.

In addition to measuring spike morphology traits, we examined some important indicators that are related to known differences in spike morphology and assimilate distribution. For example, the spike fertility index (determined as grain number per gram of spike chaff), spikelet density (spikelet number per centimeter of spike length), spikelet fertility (the ratio of fertile to total spikelet number) and floret fertility (the ratio between the maximum number of floret primordia and final grain number within individual spikelet) reveal relationships between the spike components discussed above and also demonstrate the overall effects on spike growth and development. We hypothesized that the wheat spike adapts to stress and environmental conditions by balancing the relationships between different components of the spike. Therefore, we can try to maximize the grain yield by manipulating and predicting spike morphology traits in wheat.

In this report, we first demonstrated the potential for manipulating spike morphology through a detillering experiment. Moreover, we conducted a genome-wide association study (GWAS) and genome-wide selection (GS), demonstrating the potential for genetically manipulating and predicting spike morphology in wheat. Furthermore, correlation and stepwise analyses revealed the relationship between greenhouse and field data, as well as critical spike morphometric traits for the determination of grain yield.

## Materials and Methods

### Plant material and growth conditions

Experiments were carried out at the Leibniz Institute of Plant Genetics and Crop Plant Research, Gatersleben, Germany (51°49′23″N, 11°17′13″E, altitude 112 m). The 12 hexaploid spring wheat cultivars were selected according to released years (Table S1) for detillering experiment. Control and tiller removal experiments based on the 12 wheat cultivars were conducted in the field and greenhouse simultaneously. Tillers were removed two to three times per week. Eighty plants per cultivar (forty plants for control and the other forty plants for tiller removal) were planted under field and greenhouse conditions. The 80 individuals from each cultivar were used to make sure there are enough plants for phenotypic measurement, because there are variations between individual plants within one genotype and not all the plants can be used for the phenotypic measurements. The GABI-WHEAT population contains 358 European winter and 14 spring wheat varieties. Of these, we selected 210 winter cultivars based on the different alleles at the *photoperiod* (*Ppd*) and *reduced height* (*Rht*) loci^14^ (Supplementary dataset). The genotyping work for *Ppd-D1* (chromosome 2D) and *Rht-D1* (chromosome 4D) was carried out according to the markers in previous study^15,16^. Detailed information about the genotyping process can be found in previous work^17^.

Phenotyping for the 210 winter wheat accessions was conducted in the greenhouse. The growth conditions for all the cultivars, including the 210 winter wheat accessions for GWAS, and the 12 spring wheat accessions for the detillering experiment, can be found in a previous work^3^. In brief, forty plants were planted for each cultivar. The grains were sown in 96-well trays on the same date and germinated under greenhouse conditions (photoperiod and temperature, 16:8 h, 20:16 °C, light: dark) for 14 d. Seedlings at the two- to three-leaf stage were transferred to 4 °C to vernalize for 63 d. The vernalized seedlings were transferred to a hardening stage (photoperiod and temperature, 12:12 h, 15:15 °C, light: dark) for 7 d to gradually acclimatize. Finally, all the plants were trans- planted into 0.5 l pots (one plant per pot) under controlled greenhouse conditions (photoperiod and temperature, 16:8 h, 20:6 °C, light: dark). Supplemental light (c. 250 lmol m^−2^ s^−1^ photosynthetically active radiation) was supplied with low- intensity incandescent light and plants were irrigated when required.

### Phenotypic measurements

In this study, 44 traits were measured in the 210 wheat accessions (Table 1). Spike length (cm), spike chaff per spike (g), spike dry weight (g), main stem dry weight (g), grain weight per spike (g), grain number per spike, and total and fertile spikelet numbers were measured at physiological maturity. Six plants for each cultivar were randomly selected for trait measurements. Spike length was measured without awn. The main stem was measured by the weight of the main shoot without spike but included leaves. In addition, final grain number per spikelet was measured in spikelets at three positions of the spike: apical (the third spikelet from the top of the spike), central (the spikelet in center of the spike), and basal positions (the third spikelet from the bottom of the spike). Spike fertility index, spikelet density, and fertility are determined by spikelet number per centimeter of spike length, grain number (per spike) per gram of spike chaff (per spike) at harvest^18^, and ratio of fertile and total spikelet numbers. Here, it should be noted that basal spikelets of the spike are from low-middle positions of the spike and do not include the extreme basal spikelets at the bottom of the spike. Infertile spikelets are defined as spikelets that did not set grain at all (i.e. were completely empty); whereas fertile spikelets produced at least one grain.

1 $${\rm{Spikelet}}\,{\rm{density}}=\frac{\,\text{spikelet}\,{\rm{number}}\,{\rm{per}}\,\text{spike}\,}{{\rm{spike}}\,{\rm{length}}}$$

2 $${\rm{Spike}}\,{\rm{fertility}}\,{\rm{index}}=\frac{\,\text{grain}\,{\rm{number}}\,{\rm{per}}\,\text{spike}\,}{{\rm{spike}}\,{\rm{chaff}}\,{\rm{per}}\,{\rm{spike}}}$$

3 $${\rm{Spikelet}}\,{\rm{fertility}}\,({\rm{in}}\, \% )=\frac{\,\text{fertile}\,{\rm{spikelet}}\,{\rm{number}}\,{\rm{per}}\,\text{spike}\,}{{\rm{total}}\,{\rm{spikelet}}\,{\rm{number}}\,{\rm{per}}\,{\rm{spike}}}\ast 100$$

**Table 1:** The 44 assimilate distribution and spike morphology traits in this study.

Running number	Trait	Affected plant parts
1	Grain A	main shoot
2	Grain C	main shoot
3	Grain B	main shoot
4	grain number for tillers	tiller
5	grain weight for tillers (g)	tiller
6	TKW (g)	tiller
7	grain area (mm^2^)	tiller
8	grain width (mm)	tiller
9	grain length (mm)	tiller
10	tiller number	tiller
11	tiller DW (g)	tiller
12	stem length (cm)	main shoot
13	leaf number	main shoot
14	leaf DW (g)	main shoot
15	main DW (g)	main shoot
16	spike length (cm)	main shoot
17	total spikelet number	main shoot
18	fertile spikelet number	main shoot
19	spike DW (g)	main shoot
20	main stem DW (g)	main shoot
21	grain number/spike	main shoot
22	grain weight/spike	main shoot
23	spike chaff (g)	main shoot
24	TKW (g)	main shoot
25	grain area (mm^2^)	main shoot
26	grain width (mm)	main shoot
27	grain length (mm)	main shoot
28	spikelet density	main shoot
29	spike fertility index	main shoot
30	grain weight/spike chaff	main shoot
31	spikelet fertility	main shoot
32	spike DW/main stem DW	main shoot
33	plant DW (g)	whole plant
34	grain number	whole plant
35	grain weight (g)	whole plant
36	TKW(g)	whole plant
37	grain area (mm^2^)	whole plant
38	grain width (mm)	whole plant
39	grain length (mm)	whole plant
40	harvest index	whole plant
41	tiller DW/main DW	tiller/main
42	Max.Floret A	main shoot
43	Max.Floret C	main shoot
44	Max.Floret B	main shoot

Note: A and B indicate the third spikelet from the top (apical) and bottom (base) of the spike while C (central) indicates spikelets from the middle of the spike; DW, dry weight; TKW, thousand kernel weight. Grain number A, C, B is the grain number per spikelet at different spikelet positions. Max. Floret A, C, B is the maximum number of floret primordia per spikelet at different spikelet positions. Spike chaff is the DW of spike without grains. Main DW, main stem DW, total spikelet number mean the main shoot DW, main shoot DW without spike, spikelet number per spike on the main shoot, respectively. Tiller DW/main DW, spike DW/main stem DW, grain weight/spike chaff imply the ratios between tiller DW and main shoot DW, between spike DW and main stem DW, between grain weight and spike chaff. Spike fertility index is the ratio between grain number and spike chaff of the main shoot. Spikelet fertility is the ratio between the number of fertile spikelet and total spikelet. Harvest index is obtained as the ratio between grain weight and total aboveground dry weight. The attribution of traits suggests the traits from main shoot or tillers. Grain size traits (grain length, width, area, TKW) are measured for main shoot, tillers and the whole plant (including main shoot and tillers), respectively.

The grain yield (tons/hectare) for all the 210 winter wheat accessions were determined at eight environments in the field. In this study, we reanalyzed some data to support the results with different SNP markers and approaches for different purposes.

### Plant materials and analyses of phenotypic data

The phenotypic data within each trial were analyzed using the following mixed model:

4 $$P=G+e,$$

where P were the phenotypic values in each trial, G referred to the genetic values, and e denotes the residuals. *G* and *e* were assumed to be random effects in order to estimate the repeatability. The repeatability for each experiment was calculated as: $\frac{{{\rm{V}}}_{{\rm{G}}}}{{{\rm{V}}}_{{\rm{G}}}+\frac{{{\rm{V}}}_{{\rm{e}}}}{{\rm{R}}}}$ where V_G_ was the genetic variance, V_e_ was the variance of residuals, and R referred to the number of replicates. Phenotypic values of 3 traits in different trials with repeatability estimates below 0.5 were removed from further analysis.

We used the following linear mixed model to estimate the best linear unbiased estimations (BLUEs) of the genotypes across the experiments:

5 $$P \sim G\,+T+e,$$

where P represents the phenotypic values in each trial, G referred to the genetic values, T was the effect of each trial, and e denotes the residuals. G was treated as fixed effect to estimate BLUEs and as random effect to estimate the variance component. *E* and *e* were always set as random effects. Moreover, we assumed heterogeneous error variance in each trial. The heritability was calculated as ${h}^{2}=\frac{{V}_{G}}{{V}_{G}+\frac{{V}_{e}}{OR}}$, where O was the number of average overlapped trials.

### Genome-wide association analyses

To investigate the influence of population admixture, we first used the block relaxation algorithm in the ADMIXTURE program^19^ to estimate individual ancestry proportions given an optimal number (k = 10) of hypothetical ancestral populations. Then, we performed a principal component analysis (PCA) on the wheat population of 210 cultivars. The first 10 principal components were used for subsequent analysis analyses according to the ADMIXTURE results. The PCA results did not reveal any apparent population structure among the varieties (Fig. S1). Therefore, we carried out a genome-wide association study (GWAS) with a mixed linear model using EMMA^20^. Since many associated single nucleotide polymorphism (SNP) markers were detected, we chose an overall cutoff significance level of −log_10_ (P-value) ≥ 3.0, which means that one false positive is expected in one-thousand events. It is above or close to the threshold of false discovery rate for most traits.

Marker-based estimation of heri- tability (or narrow-sense heritability, *h*^2^) was implemented based on a mixed model to compute the restricted maximum likelihood (REML)-estimates of the genetic and residual variance. Repeata- bility and heritability were calculated by the HERITABILITY R pack- age^21^.

### Genome-wide prediction

We used two genome-wide prediction models in combination with five-fold cross validation in order to evaluate the accuracy to predict genetic values for untested genotypes. The first model exploits additive effects:

6 $$y=\mu +{g}_{a}+e,({\rm{Model}}\,{\rm{BLUP}}\_{\rm{A}}).$$

The second model use additive and additive time additive effects:

7 $$y=\mu +{g}_{a}+{g}_{aa}+e,(\text{Model}\,{\rm{BLUP}}\_\text{AxA}),$$

where *y* was the vector of phenotypic records, *μ* referred to the overall mean, *g*_*a*_ were the additive genetic effect and *g*_*aa*_ were the additive × additive genetic effects. We assumed that *μ* is a fixed effect, $e\sim N(0,I{\sigma }_{e}^{2})$, ${g}_{a}\sim N(0,{G}_{a}{\sigma }_{a}^{2})$, *and* ${g}_{aa}\sim N(0,{G}_{aa}{\sigma }_{aa}^{2})$, where the matrices *G*_*a*_, and *G*_*aa*_ are the relationship matrices corresponding to additive and additive × additive epistatic genetic effects. Details of the implementation of the models have been described in Zhao *et al*. (2015).

## Results

### Modifying spike morphology through manipulation of assimilate partitioning

**Figure 1:**
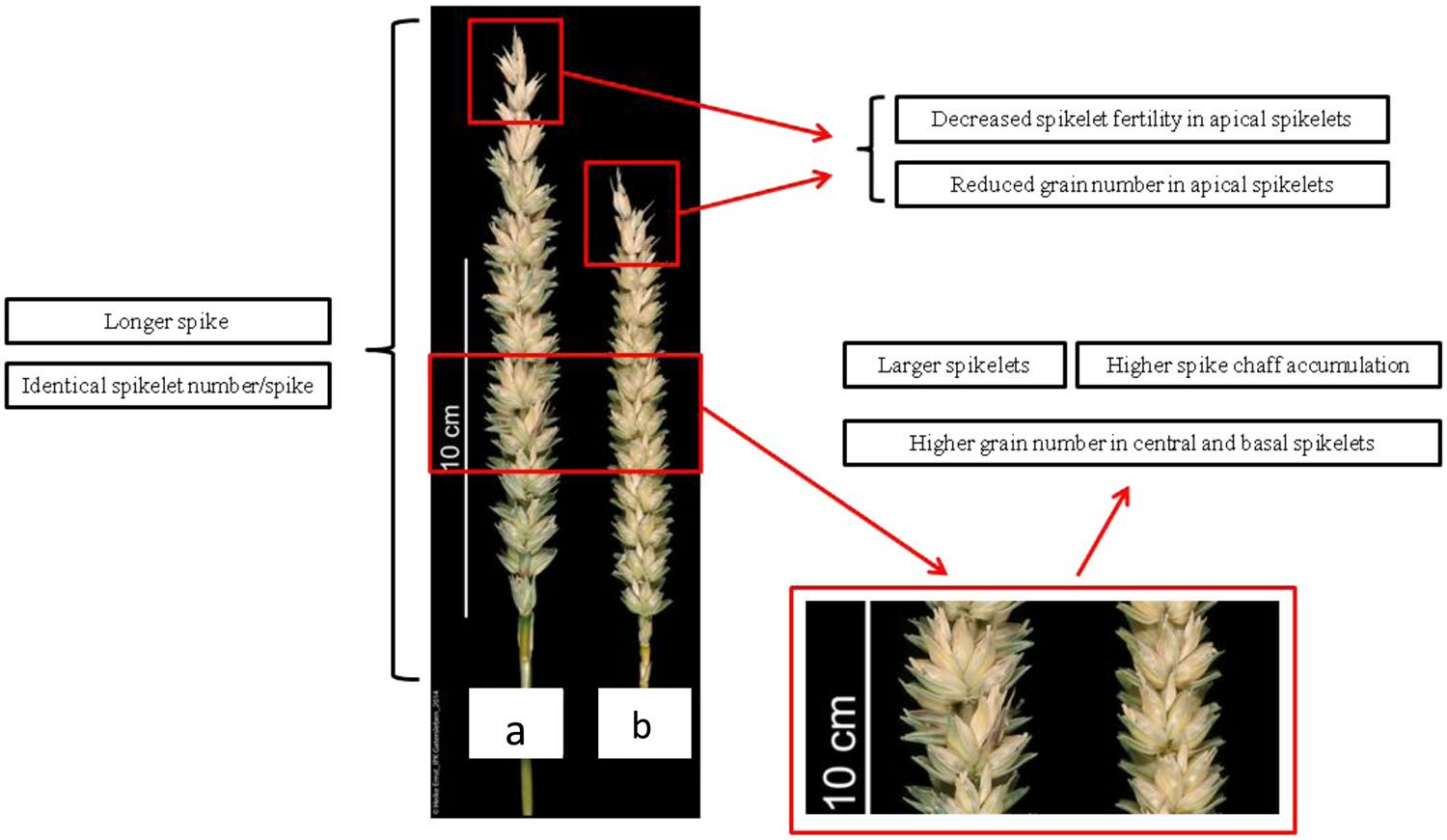
The spikes (three weeks after anthesis) in detillering (**a**) and control (**b**) treatments in the genotype ‘Breustedt’s Lera’. The traits under detillering treatment are described compared to control.

We conducted a detillering experiment to examine the potential of spike morphology manipulation by regulating allocation distribution (Fig. 1, Tables 2 and S2–8). Under field and greenhouse growth conditions, the average of spikelet density (spikelet number per centimeter of spike length) was significantly decreased by tiller removal in all the 12 cultivars (Fig. 1, Tables S2 and S3). Detillering lead to a substantial decrease of spikelet density, because tiller removal had no significant effect on total spikelet number per spike, spike length was significantly increased by tiller removal in all the 12 genotypes compared with control under both greenhouse growth conditions (Fig. 1, Tables S2 and S3).

**Table 2:** Grain number per spike, spike chaff per spike (g), and spike fertility index in 12 cultivars under control and tiller removal treatments in the greenhouse at harvest.

Cultivar	Grain number per spike		Spike chaff per spike		Spike fertility index	
	Control	Detillering	Control	Detillering	Control	Detillering
1- Adlung’s Alemannen	57.83 ± 6.52a	50.67 ± 10.93a	1.02 ± 0.13b	1.30 ± 0.10a	56.81 ± 2.11a	39.13 ± 7.82b
2- NOS Nordgau	52.00 ± 6.23a	52.40 ± 13.16a	0.61 ± 0.06b	0.98 ± 0.14a	85.14 ± 6.19a	53.37 ± 9.33b
3- Peragis Garant	44.00 ± 5.29a	52.60 ± 16.62a	0.64 ± 0.09b	0.83 ± 0.21a	69.89 ± 7.98a	62.54 ± 6.96a
4- Heine’s Peko	39.33 ± 3.08a	34.50 ± 10.63a	0.83 ± 0.10b	1.19 ± 0.17a	47.86 ± 3.69a	28.57 ± 6.24b
5- Hohenheimer Franken II	31.00 ± 3.95a	28.40 ± 6.39a	0.67 ± 0.12b	1.01 ± 0.11a	47.80 ± 11.77a	28.02 ± 4.15b
6- Probat	52.83 ± 9.15a	41.50 ± 6.66a	0.80 ± 0.12b	1.15 ± 0.15a	69.33 ± 13.09a	36.70 ± 8.95b
7- Breustedt’s Lera	41.00 ± 7.94a	47.33 ± 16.07a	0.63 ± 0.10b	1.21 ± 0.13a	64.69 ± 6.10a	40.99 ± 10.88b
8- Arin	43.67 ± 5.28b	64.67 ± 10.60a	0.61 ± 0.09b	0.84 ± 0.11a	72.10 ± 6.18a	76.72 ± 7.13a
9- Kolibri	44.00 ± 5.44b	67.67 ± 4.76a	0.72 ± 0.05b	1.19 ± 0.08a	64.04 ± 4.48a	56.84 ± 3.38b
10- Ralle	45.20 ± 3.35b	57.33 ± 10.52a	0.70 ± 0.07b	1.03 ± 0.10a	65.13 ± 3.64a	55.67 ± 6.48b
11- Nandu	46.67 ± 6.35b	66.50 ± 5.68a	0.63 ± 0.11b	1.16 ± 0.15a	74.12 ± 3.55a	58.02 ± 5.63b
12- Fasan	45.77 ± 12.47b	65.17 ± 11.97a	0.57 ± 0.14b	0.98 ± 0.15a	80.05 ± 5.58a	66.89 ± 8.17b
Average	45.33 ± 9.16b	53.34 ± 15.84a	0.70 ± 0.16b	1.07 ± 0.19a	66.45 ± 12.99a	51.26 ± 16.44b

Data are presented as the mean ± SD, n = 6; different letters per trait indicate significant differences between control and treated plants (*p* < 0.05).

Detillering also considerably decrease the average of spike fertility index (grain number per gram of spike chaff) for all the 12 cultivars. The combined influence on grain number per spike and spike chaff per spike resulted in a marked decreases in spike fertility index (with one exception, cv. Arin) in the field and greenhouse (Tables 2 and S4). The average of spikelet fertility rate (the ratio of fertile and total spikelet number) for all the 12 cultivars in control plants was considerably higher than in detillered plants (Tables S5 and S6).

Here, we found that final grain number per spikelet was increased at central or basal spikelets by tiller removal in greenhouse and field conditions (Tables S7 and S8), which may have resulted in less final grain number per spikelet at apical spikelet positions, possibly due to the competition between spikelets at different positions. At apical spikelets, especially in the greenhouse, there were large variations in the grain number per spikelet (Table  S7, S8), mainly because of completely infertile spikelets. Infertile spikelets set no grains, producing a grain number per spikelet of zero. The nearly infertile spikelets only set one or two grains per spikelet.

### Shared QTLs between assimilate distribution and spike morphology

Based the 210 winter wheat accessions, narrow sense heritability showed a broad range from 0.16 to 0.93. Of all 44 traits, 88.63% (39 traits) displayed narrow-sense heritability (*h*^2^) above 0.4, and 63.63% (28 traits) traits showed a *h*^2^ over 0.7 (Table S9), providing the genetic basis for the GWAS.

We conducted GWAS of all the 44 traits to determine the associated SNP markers that can be used to modify the spike morphology (Supplementary Dataset). Based on the GWAS results, we further assessed the shared QTLs between assimilate partitioning (e.g. tiller DW, main shoot DW, main stem DW, Table 1) and spike morphology (e.g. spike length, spikelet density, Table 1), since the main goal of this study was to investigate how the assimilate flow regulates spike morphology.

As shown in Table 3, shared QTLs on 2A (64.3 cM), 5A (5.69 cM), 5B (166.5 cM), 6B (49.0 cM) and 7B (135.7 cM) indicated the involvement of main shoot DW, main stem DW, ratio between tiller DW and main shoot DW, ratio between spike DW and main stem DW, and spike chaff in determination of spike length. It suggested that the assimilate partitioning between spike and stem as well as between tiller and main shoot, affected spikelet size/spike chaff which further lead to the modification of spike length. The association of grain number per spike on the main shoot with main stem DW, ratio between spike DW and main stem DW and spike chaff was revealed by the QTLs on 1A (26.1 cM), 1B (46.0 cM) and 7B (59.1 cM). It implied roles of assimilate allocation between spike DW and main stem DW, and spike chaff and grains in determining grain number per spike. As expected, the spike chaff per spike is associated with the ratio of tiller DW and main shoot DW, and the ratio between spike DW and main stem DW, which is indicated by shared QTLs on 1B (46.0 cM), 2B (87.5 cM) and 4A (156.3 cM). Spike fertility index can be influenced by the ratio between tiller DW and main DW, and the ratio between grain weight and spike chaff, which is revealed by 7A (1.6 cM, 89.8 cM). The shared QTLs on 3A (59.8 cM, 61.4 cM), 5B (45.8) and 7A (1.6 cM) indicated the importance of assimilate distribution between tiller and main shoot, grain and spike chaff in the improvement of spikelet fertility. Furthermore, grain number per spikelet at apical (A, the third spikelet from the top) and central (C, in the center of the spike) spikelet can be determined by the assimilate partitioning between tiller DW and main DW, spike DW and main stem DW, grains and spike chaff, which is implied by QTLs on 1B (41.3 cM), 2A (57.1 cM, 64.3 cM), 3A (59.0 cM, 59.8 cM) and 7A (13.4 cM).

**Table 3:** SNP markers associated with assimilate allocation and spike morphology traits.

Traits	Main DW	Tiller DW/main DW	Main stem DW	Spike DW/main stem DW	Grain weight per spike	Spike chaff	Grain weight/spike chaff
Total spikelet number				2A, 64.3			
Spike length	5B, 166.5	5A, 11.0	5B, 166.5	2A, 64.3		6B, 49; 7B, 135.7	
Grain number per spike			7B, 59.1	1A, 26.1		1B, 46	
Spike chaff		2B, 87.5; 4A, 156.3		1B, 46			
Spike fertility index		7A, 1.6					7A, 89.8
Spikelet fertility		3A, 59.8; 7A, 1.6				5B, 45.8	3A, 61.4
Grain number (A)		2A, 57.1; 3A, 59.0		2A, 64.3			3A, 59.8
Grain number (C)		7A, 13.4		1B, 41.3	1B, 41.3	1B, 41.3	

Note: A and C indicate the third spikelet from apical position and the spikelet in central position of the spike, respectively; DW, dry weight; TKW, thousand kernel weight. Grain number is the grain number per spikelet. Spike chaff is the DW of spike without grains on the main shoot. Main DW, main stem DW, total spikelet number mean the main shoot DW, main shoot DW without spike, spikelet number per spike on the main shoot, respectively. Tiller DW/main DW, spike DW/main stem DW, grain weight/spike chaff imply the ratios between tiller DW and main shoot DW, between spike DW and main stem DW, between grain weight and spike chaff. Spike fertility index is the ratio between grain number and spike chaff of the main shoot. Spikelet fertility is the ratio between the number of fertile spikelet and total spikelet. All data are from the main shoot, except data that have been named by tiller. SNP, single nucleotide polymorphism.

We genotyped the *Rht-D1* (Chromosome 4D) in the 210 wheat accessions and detected that allelic variants at *Rht-D1* influence spike morphology traits. The dwarf allele of *Rht-D1* significantly increased grain number per spikelet at apical positions of spike (grain A, P < 0.001); but it did not show effects on central and basal positions of spike (grain C, B). The dwarf allele significantly decreased spike DW of main shoot (P < 0.05), which may attributable to its effects on grain weight. Since the dwarf allele markedly decreased grain size (grain area, width, length and TKW, all P < 0.001); but has no obvious effects on spike chaff, it significantly increased the spike fertility index (ratio between grain number and spike chaff).

### Correlation between traits in field (grain yield) and greenhouse

**Figure 2:**
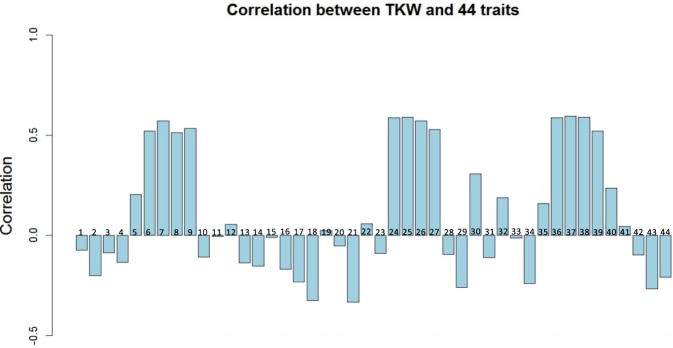
Cross-validated prediction abilities of genome-wide prediction exploiting additive (Blup_A) or additive and additive × additive effects (Blup_AxA) for all 44 traits. The traits corresponding to each number can be found in Table 1.

**Figure 3:**
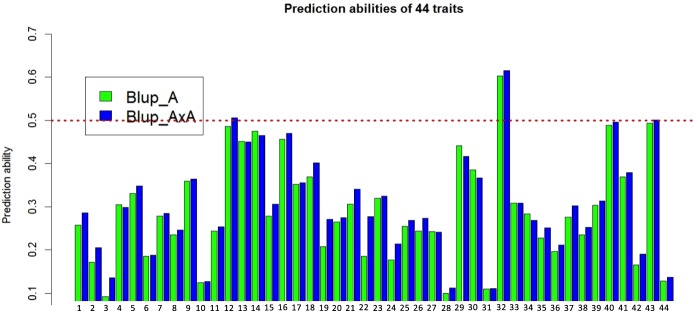
Correlations between TKW in field and the 44 traits (spike morphology and assimilate partitioning traits) in greenhouse, the corresponding number of the running number is shown in Table 1.

Using the 210 winter wheat accessions, we also assessed the correlations between the 44 traits in the greenhouse with TKW and grain yield (ton per hectare) in the field to see the relationships between yield-related traits between the field and greenhouse (Figs 2 and S2, Tables S10 and S11). We observed that individual grain area, grain width, length, TKW in the greenhouse displayed the strongest correlations (>0.5 Pearson correlation coefficient) with TKW in the filed (Fig. 3, Table S10). It suggests a the high stability of grain size between field and greenhouse. The associations of the 44 traits with grain yield in the field are not as strong as TKW (Fig. S2, Table S11). The ratio between tiller DW and main shoot DW displays the closest positive connection with grain yield in field, indicating that similar DW between tillers and main shoot (low differences between tiller DW and main shoot DW) is helpful to the improvement of grain yield (Fig. S2, Table S11). In addition, grain number per spike, grain weight per spike, tiller number, tiller DW and ratio between spike DW and stem DW also exhibited relatively strong connections with grain yield in the field (Fig. S2, Table S11), implying that not only the tiller growth but also assimilate distribution between spike and stem is beneficial to the improvement of grain yield. This is supported by the strong negative correlation between grain yield and stem DW, which suggests that reducing assimilate distribution to the stem is helpful for the improvement of grain yield. Moreover, leaf number and DW show close correlation with grain yield, suggesting that the superabundant leaf number and DW may also compete assimilates with the spike.

### Stepwise Regression for yield and TKW in field

Using the data from the 210 winter wheat accessions, the 44 traits in this study were subjected to a stepwise analysis to determine the significant variables contributing to the variation of TKW and grain yield (Tables 4 and 5). Results suggested that the most important variables contributing to TKW (field)) were TKW, main stem DW, ratio between grain weight and spike chaff, grain number per spike, leaf number in greenhouse, which can explain 27.20%, 12.42%, 12.29%, 11.63% and 4.19% of the seen variation for TKW in the field, respectively (Table 4). The effects of all the five traits on grain yield were significant (TKW P = 4.09 × 10^−9^ < 0.001, main stem DW P = 1.65 × 10^−5^ < 0.001, ratio between grain weight and spike chaff P = 1.79 × 10^−5^ < 0.001, grain number per spike P = 2.80 × 10^−5^ < 0.001, leaf number P = 0.008 < 0.01), the effects of TKW and the ratio between grain weight and spike chaff were positive, but the effects of grain number per spike, leaf number, main stem DW were negative (Table 5). The positive and negative effects can be explained by the tradeoff between grain size and number as well as the competition for assimilates between spike and main stem.

**Table 4:** Stepwise regression for TKW in the field based on the 44 traits in the greenhouse.

Yield component	Percentage of square	Effect value	Fisher test value
TKW (tillers)	27.20	0.05336	4.09E-09***
Stem DW (main shoot)	12.42	−4.34451	1.65E-05***
Grain weight/spike chaff (main shoot)	12.29	2.24634	1.79E-05***
Grain number for tillers (tillers)	11.63	−1.4021	2.80E-05***
Leaf number (main shoot)	4.19	−0.15695	0.008125**
Total	67.73		

Note: the traits from tillers or main shoot are indicated in parentheses. Grain weight/spike chaff is the ratio between spike weight and spike chaff on the main shoot. TKW, thousand kernel weight. *, **, *** suggest the significance of Fisher test value on 0.05, 0.01, 0.001 level, respectively.

**Table 5:** Stepwise regression for grain yield in the field based on the 44 traits in the greenhouse.

Yield components	Percentage of square	Effect value	Fisher test value
Grain weight/spike chaff (main shoot)	13.84	0.25361	0.000571***
Leaf number (main shoot)	11.31	−0.14824	0.001676**
Grain length (main shoot)	5.22	0.14363	0.029014*
Grain width (tillers)	4.98	−0.22353	0.032889*
Total	35.35		

Note: individual grain width means the grains from tillers; the other three traits are from main shoot. Grain weight/spike chaff is the ratio between spike weight and spike chaff on the main shoot. *, **, *** suggest the significance of Fisher test value on 0.05, 0.01, 0.001 level, respectively.

The most important variables contributing to grain yield in the field were the ratio between grain weight and spike chaff, leaf number on the main shoot, grain length and grain width, which can explain 13.84%, 11.31%, 5.22% and 4.98% of the variation for TKW in the field, respectively (Table 5). The effects of grain width and leaf number were negative, while the effects of grain length and the ratio between grain weight and spike chaff are positive (Table 5). It appears that long and thin grains are beneficial for grain setting based on the negative effects of grain width and positive effects of grain length. The positive effects of the ratio between grain weight and spike chaff indicates increased assimilates allocation towards grains improves grain yield in wheat.

In summary, in this study two traits (leaf number on the main shoot and ratio between grain weight and spike chaff) appeared to closely influence to both grain yield and TKW; while 35.35% of grain yield variation and 67.73% of the TKW variation in the field can be explained by the traits with significant effects in the greenhouse (Tables 4 and 5).

### Prediction ability of assimilate distribution and spike morphology traits

The correlation between the 44 traits (assimilate distribution and spike morphology traits) and the stepwise regression analysis suggest that grain yield and TKW are closed associated with assimilate distribution and spike morphology traits. Therefore, it is necessary to know if it is reliable to predict these traits using the SNP markers and show the prediction ability. In the 210 winter wheat accessions, the prediction ability for each trait was estimated as the Pearson correlation coefficient between the predicted values and the BLUEs. We observed moderate to high prediction ability ranging from 0.10 to 0.62 across the 44 traits. The model with additive  ×  additive interactions was on average 2% more accurate than the standard additive model (Fig. 3).

Most of assimilate partitioning traits (e.g. tiller DW, harvest index, spike fertility index) demonstrated high prediction abilities (>0.5). For the determination of spike morphology, some traits (e.g. total and fertile spikelet number, spike length) displayed relatively high prediction abilities (0.3–0.5), while others (e.g. spikelet fertility, spikelet density) exhibited relatively low prediction abilities (<0.2). Interestingly, it was detected that prediction ability for maximum number of floret primordia per spikelet (max. floret) is quite high in central spikelet (~0.5), but it is relatively low in apical and basal spikelets (<0.2), suggesting the independence of genetic regulation for max. floret at the three spikelet positions in this study.

## Discussion

### Interaction among spike morphology traits in wheat

The evident effects of tiller removal on spikes were mainly on spike length and chaff, grain number per spike, grain number per spikelet (mainly central and basal), and fertile spikelet number.

Final grain number per spikelet at central and basal spikelets of the spike was increased, while it was decreased at apical positions (S7, S8). Previous studies showed that spikelet removal resulted in a higher grain number per spikelet^10,12^. We also found that an increase in grain number per spikelet (central and basal spikelets) after tiller removal decreased fertile spikelet number per spike. Final grain number per spikelet at apical spikelets showed large variation and the apical spikelet have a lower chance to set grain compared with central and basal spikelets of the same spike, which is consistent with previous work^22,23^.

Fertile spikelet number was decreased, but total spikelet (infertile + fertile spikelet) number was unchanged (Tables S7 and S8), indicating that the decrease in fertile spikelet number per spike is mainly caused by the increase of infertile spikelets at the apical part of the spike in detillered plants.

Grain number per spike, spike length, and spike chaff were consistently increased in detillered plants (Tables 2 and S4). Consequently, spike fertility index, spikelet density and fertility were all markedly decreased by tiller removal. Grain number per spikelet were increased at the central and basal spikelets, but decreased at apical spikelets (Tables S7 and S8); hence fertile spikelet number was decreased (Tables S5 and S6). The combined influence of these four traits on grain number per spike is as follows: the increased grain number per spikelet at central and basal spikelets did not only compensate the loss caused by decreased grain number per spikelet at apical spikelets, but also finally improved the overall grain number per spike (Tables 2 and S4). The increase of chaff per spike was attributed to the increase of spikelet and floret organ size (i.e. for example glumes, lemma, palea) and spike length (predominantly rachis internode length; Fig. 1). The increase of grain number per spikelet resulted in the increase of spikelet fertility and size at central and basal spikelets (Fig. 1). We found increased spike length and unchanged total spikelet (fertile + infertile spikelets) numbers in this experiment, consistent with previous work in the wheat *tin* (*tiller inhibition*) mutant^5,24^.

### Assimilates distribution on spike morphology traits

Tillering is a core component of plant architecture^25^. Varying tiller number may produce significant differences in spike morphology^26–28^. A major *tiller inhibition* (*tin*) gene (a wheat cellulose synthase-like gene) can restrict tiller production^4,25,29,30^. Higher kernel weights in *tin* lines were realized even with the greater kernel number per spike as there was little difference in grain size^4,31^.

The effects of the *tin* gene on spike morphology traits were supported by the results in this report. Here, we observed that the ratio between tiller DW and main shoot DW shares SNP markers with spike chaff per spike, spike fertility index, spikelet fertility and grain number per spikelet. These shared markers suggest the close association of spike morphology traits with assimilate distribution between tiller and main shoot.

The utilization of dwarfing genes *Rht-B1b* and *Rht-D1b* in wheat significantly increased grain yield, because of their effects on grain number and size. In this study, we found that the ratio between spike DW and main stem DW shares SNP markers with spikelet number per spike, spike length, grain number per spike, grain number per spikelet, and spike chaff per spike. This implies the connection of assimilate partitioning between spike and stem with spike morphology traits, which is consistent with the effects of allelic variants of *Rht* loci on spike morphology.

### Important traits for the determination of grain yield and size

As expected, the ratio between grain weight and spike chaff can positively and significantly influence both grain yield and TKW. The ratio reveals the assimilates allocation between grain and the spike bracts (glume, lemma, and palea) which are the main photosynthesis parts of wheat spike^32,33^. Photosynthesis by spikes may make a larger contribution to final grain yield than flag leaves when drought develops during grain filling^32,34,35^. The length and width of glume and lemma can significantly affect grain size in wheat^36^.

Although photosynthesis by wheat spikes (glume, lemma, palea) makes a large contribution to final grain yield^37–39^, the excessive growth of glume, lemma and palea will increase their competition strength for assimilates, which may further reduce the assimilates allocation to grain growth and finally decrease final grain yield. This may explain the positive contribution of the ratio between grain weight and spike chaff to grain yield.

Leaf development is critical for the determination of grain yield, since the leaves are the main photosynthetic part^40–43^. Also, maintaining green leaves during grain filling possibly leads to increased grain yield, since the duration of leaf senescence during grain filling has been shown to affect both carbon and nitrogen partitioning and remobilization^44–47^. However, the redundant leaves may result in improved competition strength for assimilates and they similarly decrease of assimilate allocation to spike growth. This can explain the negative contribution of leaf number to grain yield.

## Electronic supplementary material

Table S1-S9, Figure S1, S2

Dataset 1
