# Supplementary material for: Manipulation and prediction of spike morphology traits for the improvement of grain yield in wheat
Source: Sci Rep. 2018 Sep 26;8:14435. doi: 10.1038/s41598-018-31977-3 (PMC6158183; doi:10.1038/s41598-018-31977-3)
Supplement: Supplementary file 1 — Table S1-S9, Figure S1, S2 [file 41598_2018_31977_MOESM1_ESM.pdf]

Manipulation and prediction of spike morphology traits for improvement of grain yield in wheat

Zifeng Guo[1], Yusheng Zhao[2], Marion S. Röder[3], Jochen C. Reif[2], Martin W. Ganal[4], Dijun Chen[5], Thorsten Schnurbusch[1*]

1 Independent HEISENBERG Research Group Plant Architecture, Leibniz Institute of Plant Genetics and Crop Plant Research, 06466 Gatersleben, Germany

2 Research Group Quantitative Genetics, Department of Breeding Research, Leibniz Institute of Plant Genetics and Crop Plant Research, 06466 Gatersleben, Germany

3 Research Group Gene and Genome Mapping, Department of Breeding Research, Leibniz Institute of Plant Genetics and Crop Plant Research, 06466 Gatersleben, Germany

4 TraitGenetics GmbH, 06466 Gatersleben, Germany

5 Research Group Image Analysis, Leibniz Institute of Plant Genetics and Crop Plant Research, 06466 Gatersleben, Germany

* To whom correspondence should be addressed: E-mail: thor@ipk-gatersleben.de, 00490394825341

Table S1. The released years of the 12 wheat cultivars for detillering experiments.

Running number	cultivar name	Year of release
1	Adlungs Alemannen	1931
2	NOS Nordgau	1933
3	Peragis Garant	1946
4	Heines Peko	1947
5	Hohenheimer Franken II	1951
6	Probat	1953
7	Breustedts Lera	1959
8	Arin	1962
9	Kolibri	1966
10	Ralle	1980
11	Nandu	1988
12	Fasan	1997

Table S2. Spikelet number per spike, spike length (cm), and spikelet density in 12 cultivars under control and tiller removal treatments in the greenhouse at harvest.

Cultivar	Total spikelet number /spike		Spike length		Spikelet density	
	Control	Detillering	Control	Detillering	Control	Detillering
1- Adlung's Alemannen	20.50±2.43a	20.67±1.03a	11.88±0.50a	12.82±1.72a	1.73±0.21a	1.64±0.29a
2- NOS Nordgau	23.33±1.21a	22.00±1.22a	11.60±0.66b	13.48±0.88a	2.01±0.08a	1.64±0.13b
3- Peragis Garant	20.17±1.60a	21.00±2.10a	10.97±0.40a	12.50±1.74a	1.84±0.17a	1.70±0.23a
4- Heine's Peko	24.83±1.33a	24.33±2.25a	10.62±1.08a	11.48±0.84a	2.35±0.17a	2.12±0.20a
5- Hohenheimer Franken II	20.50±0.84a	20.67±0.82a	10.94±0.43b	12.76±0.46a	1.85±0.08a	1.60±0.04b
6- Probat	28.33±1.51a	27.25±3.59a	12.17±0.70a	12.98±1.45a	2.33±0.11a	2.11±0.26a
7- Breustedt's Lera	26.83±0.98a	25.60±2.30a	11.13±0.78a	12.76±1.33a	2.42±0.17a	2.08±0.30a
8- Arin	22.83±0.75a	22.00±1.41a	10.48±0.41b	12.45±0.31a	2.18±0.12a	1.77±0.08b
9- Kolibri	23.00±0.89a	22.50±1.05a	10.77±0.95b	12.57±0.70a	2.15±0.22a	1.79±0.08b
10- Ralle	22.80±1.30a	23.67±1.03a	11.36±0.46b	12.47±0.65a	2.01±0.12a	1.90±0.15a
11- Nandu	23.17±1.72a	24.33±1.86a	11.32±0.92b	13.40±0.39a	2.06±0.23a	1.82±0.13a
12- Fasan	25.33±1.97a	27.00±0.63a	10.13±0.77b	12.77±0.96a	2.50±0.14a	2.13±0.18b
Average	23.48±2.79a	23.29±2.69a	11.11±0.87b	12.68±1.09a	2.13±0.28a	1.86±0.25b

Data are presented as the mean±SD, n=6. Different letters per trait indicate significant differences between control and treated plants (p=0.05).

Table S3. Spikelet number per spike, spike length (cm), and spikelet density in 12 cultivars under control and tiller removal treatments in the field at harvest.

Cultivar	Spikelet number /spike		Spike length		Spikelet density	
	Control	Detillering	Control	Detillering	Control	Detillering
1- Adlung's Alemannen	19.67±1.21a	19.00±0.89a	11.05±0.62b	13.68±0.99a	1.78±0.11a	1.39±0.09b
2- NOS Nordgau	22.50±2.07a	21.33±1.75a	11.33±1.05b	12.70±1.00a	1.99±0.21a	1.69±0.16b
3- Peragis Garant	20.00±1.10a	19.33±1.51a	12.12±1.45b	13.22±0.74a	1.66±0.15a	1.46±0.08b
4- Heine's Peko	23.17±1.47a	24.05±1.76a	12.05±1.22b	14.05±1.07a	1.93±0.18a	1.75±0.11b
5- Hohenheimer Franken II	18.67±0.82a	19.00±1.41a	10.82±1.16b	13.02±0.96a	1.74±0.15a	1.46±0.07b
6- Probat	25.00±0.89a	21.67±2.52a	12.52±1.40a	12.33±1.17a	2.02±0.20a	1.66±0.15b
7- Breustedt's Lera	23.50±1.76a	23.00±0.00a	12.05±1.49a	12.20±1.44a	1.96±0.17a	1.90±0.22a
8- Arin	21.67±2.42a	22.50±1.00a	11.92±1.17b	13.48±0.31a	1.82±0.08a	1.67±0.10b
9- Kolibri	20.67±1.03a	21.17±1.72a	10.73±1.12b	12.18±1.25a	1.94±0.21a	1.74±0.13b
10- Ralle	21.17±1.83a	22.20±0.84a	11.18±1.78b	12.74±0.34a	1.92±0.23a	1.74±0.05b
11- Nandu	23.00±1.26a	23.40±0.55a	11.10±0.76b	14.12±1.11a	2.08±0.20a	1.66±0.12b
12- Fasan	23.83±0.98a	24.17±1.60a	11.51±1.02b	12.47±1.49a	2.08±0.14a	1.95±0.17a
Average	21.90±2.30a	21.66±2.34a	11.53±1.26b	13.07±1.15a	1.91±0.21a	1.67±0.20b

Data are presented as the mean±SD, n=6; Different letters per trait indicate significant differences between control and treated plants (p=0.05).

Table S4. Grain number per spike, spike chaff per spike (g), and spike fertility index in 12 cultivars under control and tiller removal treatments in the field at harvest.

Cultivar	Grain number /spike		Spike chaff/spike		Spike fertility index	
	Control	Detillering	Control	Detillering	Control	Detillering
1- Adlung's Alemannen	51.17±1.83b	61.5±6.72a	0.66±0.14b	1.73±0.10a	80.41±16.36a	52.59±5.80b
2- NOS Nordgau	61.00±13.28b	77.17±20.25a	0.55±0.09b	0.87±0.16a	110.02±9.55a	90.92±25.86b
3- Peragis Garant	48.00±18.21b	49.00±7.07a	0.67±0.15b	0.85±0.18a	69.78±13.90a	59.19±10.90b
4- Heine's Peko	47.33±7.81b	60.50±10.45a	0.86±0.17b	1.06±0.19a	57.83±21.26a	57.55±7.06a
5- Hohenheimer Franken II	38.33±3.33a	38.50±7.87a	0.69±0.12b	0.98±0.08a	57.25±11.63a	39.59±8.10b
6- Probat	75.17±9.20a	--	0.74±0.11a	--	102.25±11.33a	--
7- Breustedt's Lera	75.40±14.72a	54.33±17.67b	0.70±0.07a	0.66±0.10a	107.29±17.65a	82.84±28.53b
8- Arin	82.17±10.20a	65.25±18.57b	0.83±0.23a	0.86±0.16a	103.92±20.56a	76.17±12.37b
9- Kolibri	50.67±9.09b	62.00±11.01a	0.71±0.12a	0.86±0.17a	72.55±14.73a	73.66±13.05a
10- Ralle	47.33±7.94a	--	0.66±0.14a	--	70.32±11.83a	--
11- Nandu	59.33±12.11a	--	0.68±0.12a	--	57.25±11.62a	--
12- Fasan	77.00±10.41a	49.00±10.00b	0.82±0.11a	0.85±0.05a	93.66±6.15a	57.23±9.24b
Average	59.18±17.21a	57.35±15.84a	0.72±0.15b	1.02±0.36a	84.25±22.60a	60.48±20.95b

Data are presented as the mean±SD, n=6; different letters per trait indicate significant differences between control and treated plants (*p*=0.05). The dashed line in the table indicates the missing data.

Table S5. Total spikelet number, fertile spikelet number, and spikelet fertility (%) in 12 cultivars under control and tiller removal treatments in the greenhouse at harvest.

Cultivar	Total spikelet number/spike		Fertile spikelet number/spike		Spikelet fertility (%)	
	Control	Detillering	Control	Detillering	Control	Detillering
1- Adlung's Alemannen	20.50±2.43a	20.67±1.03a	20.33±2.42a	13.00±2.28b	99.21±1.94a	62.65±8.27b
2- NOS Nordgau	23.33±1.21a	22.00±1.22a	20.33±1.63a	15.60±2.51b	87.12±4.74a	70.69±8.07b
3- Peragis Garant	20.17±1.60a	21.00±2.10a	17.00±1.41a	14.67±5.54b	84.89±11.32a	69.24±22.60a
4- Heine's Peko	24.83±1.33a	24.33±2.25a	15.83±2.23a	12.00±3.22a	63.80±8.89a	48.83±9.77b
5- Hohenheimer Franken II	20.50±0.84a	20.67±0.82a	16.00±3.24a	10.25±0.96b	77.91±16.77a	49.35±3.11b
6- Probat	28.33±1.51a	27.25±3.59a	18.50±2.51a	13.75±3.50b	65.73±11.74a	50.97±12.93a
7- Breustedt's Lera	26.83±0.98a	25.60±2.30a	16.83±3.60a	12.60±2.87b	62.44±11.44a	48.69±12.67a
8- Arin	22.83±0.75a	22.00±1.41a	15.33±1.21b	19.00±2.61a	67.17±5.11b	86.21±8.94a
9- Kolibri	23.00±0.89a	22.50±1.05a	20.33±2.34a	20.50±2.26a	88.39±9.39a	90.93±6.66a
10- Ralle	22.80±1.30a	23.67±1.03a	21.00±1.58a	20.83±2.56a	92.11±4.69a	88.04±9.96a
11- Nandu	23.16±1.72a	24.33±1.86a	19.33±3.14a	20.83±3.76a	83.71±13.98a	85.92±15.70a
12- Fasan	25.33±1.97a	27.00±0.63a	18.17±3.54a	19.83±0.75a	71.66±12.43a	73.49±3.29a
Average	23.48±2.79a	23.29±2.69a	18.24±3.02a	16.54±4.57b	78.50±15.16a	70.81±18.52b

Data are presented as the mean±SD, n=6; different letters per trait indicate significant differences between control and treated plants (*p*=0.05).

Table S6. Total spikelet number, fertile spikelet number, and spikelet fertility (%) in 12 cultivars under control and tiller removal treatments in the field at harvest.

Cultivar	Total spikelet number/spike		Fertile spikelet number/spike		Spikelet fertility (%)	
	Control	Detillering	Control	Detillering	Control	Detillering
1- Adlung's Alemannen	19.67±1.21a	19.00±0.89a	18.83±1.47a	15.33±2.50b	95.78±4.95a	80.73±12.63b
2- NOS Nordgau	22.50±2.07a	21.33±1.75a	20.83±1.72a	20.50±2.07a	92.80±5.57a	96.01±3.73a
3- Peragis Garant	20.00±1.10a	19.33±1.51a	19.00±1.10a	18.67±2.25a	95.03±3.17a	96.39±6.70a
4- Heine's Peko	23.17±1.47a	24.50±1.76a	22.33±1.86a	21.50±1.22a	96.41±5.61a	88.25±9.57b
5- Hohenheimer Franken II	18.67±0.82a	18.60±1.14a	16.83±2.64a	13.40±2.70b	90.15±13.47a	71.68±11.50b
6- Probat	25.00±0.89a	21.67±2.52a	24.33±1.21a	18.00±1.00b	97.33±3.27a	83.49±5.35b
7- Breustedt's Lera	23.20±1.79a	23.00±0.00a	21.80±1.30a	19.33±2.08b	94.13±4.52a	84.06±9.05b
8- Arin	21.67±2.42a	22.50±1.00a	21.50±2.74a	21.50±0.58a	99.07±2.27a	95.74±5.90a
9- Kolibri	20.67±1.03a	21.17±1.72a	19.83±1.60a	20.50±1.38a	95.95±5.83a	97.07±5.28a
10- Ralle	21.17±1.83a	22.20±0.84a	19.67±2.42a	17.40±3.51b	92.73±5.31a	78.03±13.26b
11- Nandu	23.00±1.26a	23.40±0.55a	21.17±1.17a	22.60±0.55a	92.19±5.96a	96.63±3.50a
12- Fasan	23.83±0.98a	23.25±0.96a	23.33±1.63a	20.40±3.65b	97.83±3.64a	83.81±14.32b
Average	21.86±2.28a	21.53±2.25a	20.77±2.63a	19.10±3.33b	94.96±6.05a	88.19±11.85b

Data are presented as the mean±SD, n=6; different letters per trait indicate significant differences between control and treated plants (*p*=0.05).

Table S7. Infertile spikelet number per spike and grain number per spikelet in apical, central, and basal spikelets in 12 cultivars under control and tiller removal treatments in the greenhouse at harvest.

Cultivar	Infertile spikelet number/spike		Grain number/spikelet					
			Apical spikelet		Central spikelet		Basal spikelet	
	Control	Detillering	Control	Detillering	Control	Detillering	Control	Detillering
1- Adlung's Alemannen	0.17±0.41b	7.67±1.51a	2.50±0.55a	0.16±0.41b	3.67±0.52a	4.33±0.82a	3.67±0.52b	4.67±0.52a
2- NOS Nordgau	3.00±1.10b	6.40±1.67a	2.16±0.41a	0.40±0.89b	3.00±0.00a	3.60±0.55a	3.17±0.41b	3.80±0.84a
3- Peragis Garant	3.17±2.48b	6.33±4.63a	1.83±0.75a	1.83±1.60a	2.83±0.75a	2.83±1.72a	3.17±0.75a	3.50±0.84a
4- Heine's Peko	9.00±2.28b	12.33±1.97a	0.16±0.41a	0.00±0.00b	2.83±0.41a	1.50±1.64b	3.17±0.75b	3.83±0.41a
5- Hohenheimer Franken II	4.60±3.58b	10.50±0.58a	1.00±1.10a	0.00±0.00a	2.00±0.63a	1.17±1.33b	2.83±0.41b	3.33±1.21a
6- Probat	9.83±3.71b	13.50±4.73a	0.17±0.41a	0.00±0.00b	3.17±0.41a	2.00±1.63b	3.17±0.41a	3.25±0.50a
7- Breustedt's Lera	10.00±2.75b	13.00±3.59a	0.50±0.55a	0.00±0.00b	2.33±1.21a	2.40±2.07a	2.67±0.52b	3.20±1.79a
8- Arin	7.50±1.22a	3.00±1.90b	0.83±0.75b	2.17±0.75a	3.33±0.52b	4.50±0.55a	3.00±0.63b	4.00±1.10a
9- Kolibri	2.67±2.16a	2.00±1.41a	1.83±0.41b	2.83±0.41a	3.00±0.00b	4.17±0.41a	2.33±0.82b	4.33±0.52a
10- Ralle	1.80±1.10b	2.83±2.40a	1.80±0.45a	1.50±0.84b	2.40±0.55b	3.50±0.55a	2.60±0.55b	3.50±0.55a
11- Nandu	3.83±3.37a	3.50±3.94a	1.83±0.41a	1.83±0.98a	3.00±0.00b	4.50±0.55a	2.50±0.84b	4.17±0.98a
12- Fasan	7.17±3.13a	7.17±0.98a	1.50±0.84a	1.67±0.75a	2.83±0.41b	3.83±0.98a	2.67±1.37b	3.50±0.55a
Average	5.29±3.97b	6.86±4.55a	1.34±0.96a	1.04±1.20a	2.91±0.58a	3.78±0.94a	2.96±0.67b	3.85±0.85a

Data are presented as the mean±SD, n=6; different letters per trait indicate significant differences between control and treated plants (p=0.05).

Table S8. Infertile spikelet number per spike and grain number per spikelet at apical, central, and basal spikelets in 12 cultivars under control and tiller removal treatments in the field at harvest.

Cultivar	Infertile spikelet number/spike		Grain number/spikelet					
			Apical spikelet		Central spikelet		Basal spikelet	
	Control	Detillering	Control	Detillering	Control	Detillering	Control	Detillering
1- Adlung's Alemannen	0.83±0.98b	3.67±2.42a	2.50±0.55a	2.33±1.75a	3.67±0.82b	4.67±0.52a	3.50±0.55b	4.33±1.21a
2- NOS Nordgau	1.67±1.37a	0.83±0.75b	2.83±0.75a	3.00±1.55a	3.67±0.82b	4.50±0.84a	3.67±0.52b	4.50±0.84a
3- Peragis Garant	0.83±1.33b	0.67±1.21a	1.83±0.41a	2.50±1.05a	2.67±0.52b	3.33±1.03a	2.67±0.52b	3.33±1.03a
4- Heine's Peko	0.83±1.33b	3.00±2.45a	1.83±0.41a	2.00±0.63a	2.67±0.52b	3.50±0.84a	2.67±0.52b	3.33±1.03a
5- Hohenheimer Franken II	1.83±2.48b	5.20±1.92a	2.33±0.52a	0.50±0.548b	2.50±0.55b	3.33±0.52a	3.00±0.63b	3.50±0.55a
6- Probat	0.67±0.82b	3.67±1.53a	2.50±0.55a	2.25±0.97a	3.83±0.75a	4.00±1.15a	3.33±1.03a	3.50±1.29a
7- Breustedt's Lera	1.40±1.14b	3.67±2.08a	2.33±1.37a	2.00±1.73a	3.83±1.47b	4.33±0.58a	4.50±0.55a	3.33±1.53b
8- Arin	0.17±0.41b	1.00±1.41a	3.50±1.05a	3.50±1.00a	4.17±0.75a	3.75±1.26b	4.50±0.84a	3.75±0.96b
9- Kolibri	0.83±1.17a	0.67±1.21a	2.33±0.52b	2.83±0.41a	3.17±0.75b	4.17±1.17a	3.33±1.03a	3.50±1.52a
10- Ralle	1.50±1.05b	4.80±2.77a	2.17±0.41a	1.50±1.64b	3.17±0.41a	3.33±0.82a	2.83±0.75b	4.00±0.89a
11- Nandu	1.83±1.47a	0.80±0.84b	2.50±0.55b	3.00±0.00a	3.67±0.52b	4.17±0.75a	3.33±1.03a	3.17±0.98a
12- Fasan	0.50±0.84b	3.00±3.31a	2.83±0.41a	1.83±1.33b	3.83±1.17a	3.00±0.63b	4.17±1.47a	3.17±0.98b
Average	1.08±1.26b	2.46±2.44a	2.53±0.75a	2.25±1.31ba	3.47±0.90b	3.82±0.95a	3.49±1.02a	3.63±1.07a

Data are presented as the mean±SD, n=6; different letters per trait indicate significant differences between control and treated plants (p=0.05).

..

Table S9. Heritabilities of the 44 traits in the 210 winter wheat cultivars.

number	traits	attribution of traits	h^2
1	Grain A	main shoot	0.46
2	Grain C	main shoot	0.49
3	Grain B	main shoot	0.37
4	grain number for tillers	tiller	0.66
5	grain weight for tillers (g)	tiller	0.72
6	TKW (g)	tiller	0.77
7	seed area (mm^2)	tiller	0.91
8	seed width (mm)	tiller	0.88
9	seed length (mm)	tiller	0.92
10	tiller number	tiller	0.72
11	tiller DW (g)	tiller	0.72
12	stem length (cm)	main shoot	0.88
13	leaf number	main shoot	0.24
14	leaf DW (g)	main shoot	0.65
15	main DW (g)	main shoot	0.71
16	spike length (cm)	main shoot	0.81
17	total spikelet number	main shoot	0.82
18	fertile spikelet number	main shoot	0.56
19	spike DW (g)	main shoot	0.72
20	main stem DW (g)	main shoot	0.73
21	grain number/spike	main shoot	0.69
22	grain weight/spike	main shoot	0.70
23	spike chaff (g)	main shoot	0.94
24	TKW (g)	main shoot	0.71
25	seed area (mm^2)	main shoot	0.92
26	seed width (mm)	main shoot	0.90
27	seed length (mm)	main shoot	0.93
28	spikelet density	main shoot	0.80
29	spike fertility index	main shoot	0.16
30	grain weight/spike chaff	main shoot	0.17
31	spikelet fertility	main shoot	0.52
32	spike DW/main stem DW	main shoot	0.70
33	plant DW (g)	whole plant	0.76
34	grain number	whole plant	0.73
35	grain weight (g)	whole plant	0.79
36	TKW(g)	whole plant	0.83
37	seed area (mm^2)	whole plant	0.93
38	seed width (mm)	whole plant	0.92
39	seed length (mm)	whole plant	0.94
40	harvest index	whole plant	0.77
41	tiller DW/ main DW	tiller/main	0.41
42	Max.Floret A	main shoot	0.36
43	Max.Floret C	main shoot	0.42
44	Max.Floret B	main shoot	0.50

Table S10. Correlations values between TKW in the field and the 44 traits (spike morphology and assimilate partitioning traits) in the greenhouse based on the 210 winter wheat cultivars.

number	traits	attribution of traits	correlations values with TKW
1	Grain A	main shoot	-0.075
2	Grain C	main shoot	-0.201
3	Grain B	main shoot	-0.087
4	grain number for tillers	tiller	-0.136
5	grain weight for tillers (g)	tiller	0.203
6	TKW (g)	tiller	0.521
7	seed area (mm^2)	tiller	0.570
8	seed width (mm)	tiller	0.514
9	seed length (mm)	tiller	0.534
10	tiller number	tiller	-0.109
11	tiller DW (g)	tiller	-0.005
12	stem length (cm)	main shoot	0.055
13	leaf number	main shoot	-0.136
14	leaf DW (g)	main shoot	-0.152
15	main DW (g)	main shoot	-0.011
16	spike length (cm)	main shoot	-0.168
17	total spikelet number	main shoot	-0.234
18	fertile spikelet number	main shoot	-0.326
19	spike DW (g)	main shoot	0.023
20	main stem DW (g)	main shoot	-0.052
21	grain number/spike	main shoot	-0.333
22	grain weight/spike	main shoot	0.058
23	spike chaff (g)	main shoot	-0.089
24	TKW (g)	main shoot	0.586
25	seed area (mm^2)	main shoot	0.590
26	seed width (mm)	main shoot	0.573
27	seed length (mm)	main shoot	0.529
28	spikelet density	main shoot	-0.095
29	spike fertility index	main shoot	-0.260
30	grain weight/spike chaff	main shoot	0.308
31	spikelet fertility	main shoot	-0.111
32	spike DW/main stem DW	main shoot	0.188
33	plant DW (g)	whole plant	-0.013
34	grain number	whole plant	-0.241
35	grain weight (g)	whole plant	0.158
36	TKW(g)	whole plant	0.587
37	seed area (mm^2)	whole plant	0.597
38	seed width (mm)	whole plant	0.590
39	seed length (mm)	whole plant	0.521
40	harvest index	whole plant	0.235
41	tiller DW/ main DW	tiller/main	0.044
42	Max.Floret A	main shoot	-0.098
43	Max.Floret C	main shoot	-0.265
44	Max.Floret B	main shoot	-0.209

Table S11. Correlations values between grain yield (ton per hectare) in the field and the 44 traits (spike morphology and assimilate partitioning traits) in the greenhouse based on the 210 winter wheat cultivars.

number	traits	attribution of traits	correlations values with grain yield
1	Grain A	main shoot	-0.117
2	Grain C	main shoot	-0.022
3	Grain B	main shoot	-0.005
4	grain number for tillers	tiller	0.241
5	grain weight for tillers (g)	tiller	0.192
6	TKW (g)	tiller	-0.077
7	seed area (mm^2)	tiller	-0.032
8	seed width (mm)	tiller	-0.152
9	seed length (mm)	tiller	0.078
10	tiller number	tiller	0.186
11	tiller DW (g)	tiller	0.232
12	stem length (cm)	main shoot	-0.069
13	leaf number	main shoot	-0.156
14	leaf DW (g)	main shoot	-0.179
15	main DW (g)	main shoot	-0.091
16	spike length (cm)	main shoot	-0.027
17	total spikelet number	main shoot	0.014
18	fertile spikelet number	main shoot	0.013
19	spike DW (g)	main shoot	0.035
20	main stem DW (g)	main shoot	-0.183
21	grain number/spike	main shoot	0.044
22	grain weight/spike	main shoot	0.086
23	spike chaff (g)	main shoot	-0.121
24	TKW (g)	main shoot	0.107
25	seed area (mm^2)	main shoot	0.095
26	seed width (mm)	main shoot	0.053
27	seed length (mm)	main shoot	0.122
28	spikelet density	main shoot	0.040
29	spike fertility index	main shoot	0.106
30	grain weight/spike chaff	main shoot	0.062
31	spikelet fertility	main shoot	-0.112
32	spike DW/main stem DW	main shoot	0.183
33	plant DW (g)	whole plant	0.092
34	grain number	whole plant	0.138
35	grain weight (g)	whole plant	0.088
36	TKW(g)	whole plant	-0.042
37	seed area (mm^2)	whole plant	0.009
38	seed width (mm)	whole plant	-0.063
39	seed length (mm)	whole plant	0.095
40	harvest index	whole plant	0.027
41	tiller DW/ main DW	tiller/main	0.282
42	Max.Floret A	main shoot	0.079
43	Max.Floret C	main shoot	-0.028
44	Max.Floret B	main shoot	0.043

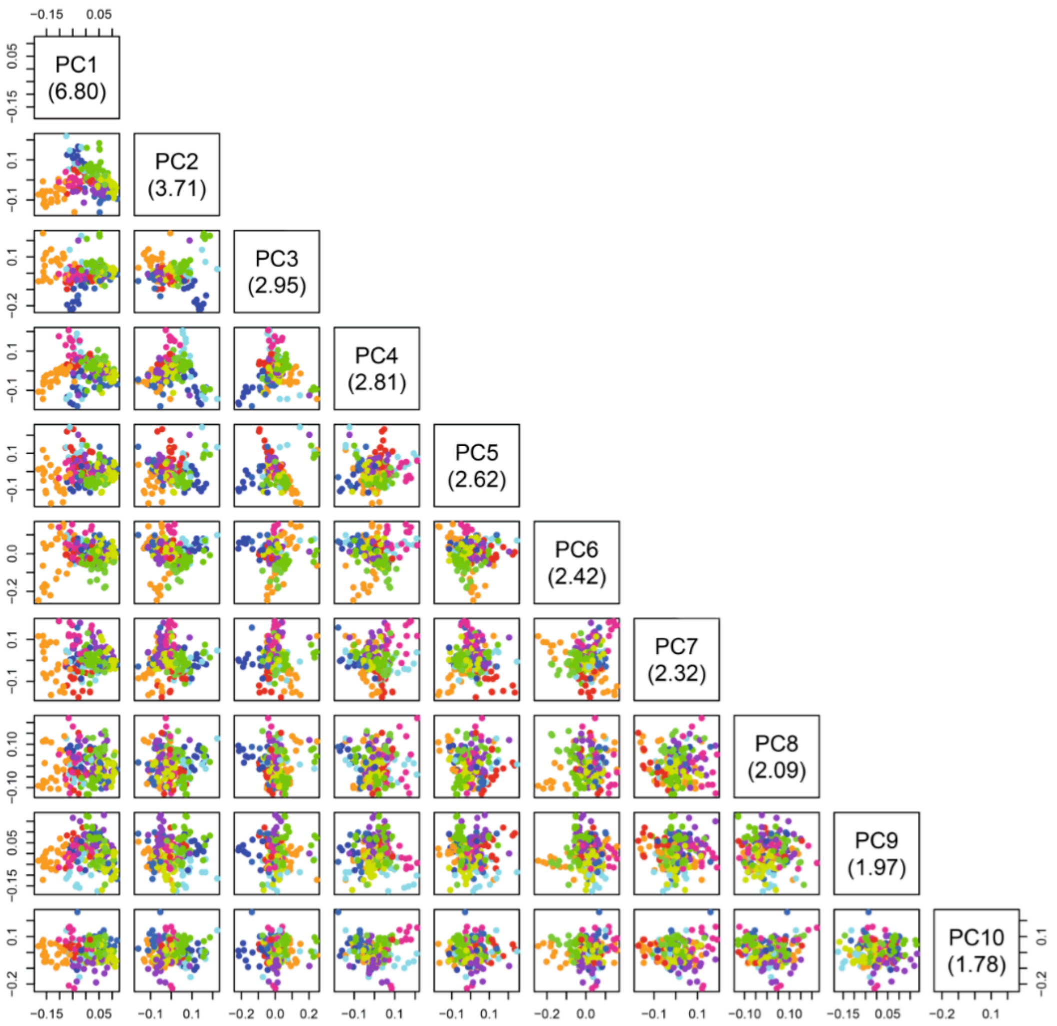

Figure S1. Genetic population structure determined by principal component analysis with SNP markers based on the 210 wheat cultivars.

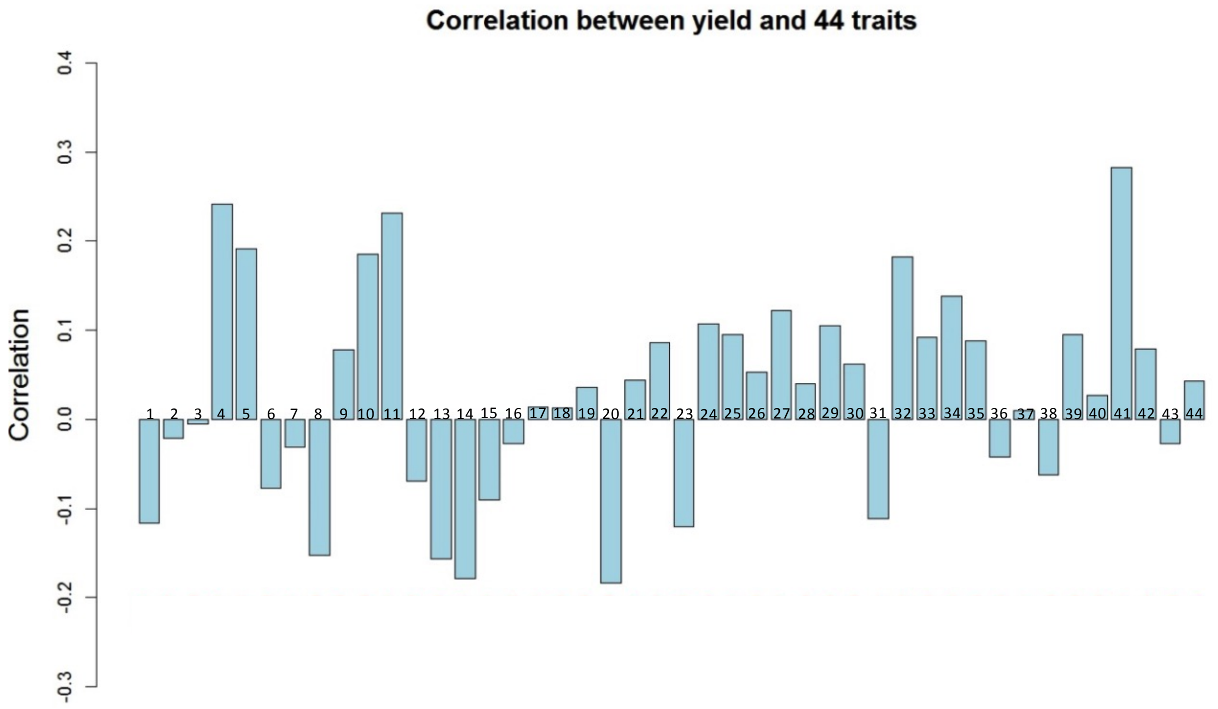

Figure S2. Correlations between grain yield in field and the 44 traits (spike morphology and assimilate partitioning traits) in greenhouse based on the 210 wheat cultivars.
